# DRL-Assisted Resource Allocation for NOMA-MEC Offloading with Hybrid SIC

**DOI:** 10.3390/e23050613

**Published:** 2021-05-14

**Authors:** Haodong Li, Fang Fang, Zhiguo Ding

**Affiliations:** 1 Department of Electrical and Electronic Engineering, The University of Manchester, Manchester M13 9PL, UK; zhiguo.ding@manchester.ac.uk; 2 Department of Engineering, Durham University, Durham DH1 3LE, UK; fang.fang@durham.ac.uk

**Keywords:** deep reinforcement learning (DRL), multi-access edge computing (MEC), resource allocation, sixth-generation (6G), user grouping

## Abstract

Multi-access edge computing (MEC) and non-orthogonal multiple access (NOMA) are regarded as promising technologies to improve the computation capability and offloading efficiency of mobile devices in the sixth-generation (6G) mobile system. This paper mainly focused on the hybrid NOMA-MEC system, where multiple users were first grouped into pairs, and users in each pair offloaded their tasks simultaneously by NOMA, then a dedicated time duration was scheduled to the more delay-tolerant user for uploading the remaining data by orthogonal multiple access (OMA). For the conventional NOMA uplink transmission, successive interference cancellation (SIC) was applied to decode the superposed signals successively according to the channel state information (CSI) or the quality of service (QoS) requirement. In this work, we integrated the hybrid SIC scheme, which dynamically adapts the SIC decoding order among all NOMA groups. To solve the user grouping problem, a deep reinforcement learning (DRL)-based algorithm was proposed to obtain a close-to-optimal user grouping policy. Moreover, we optimally minimized the offloading energy consumption by obtaining the closed-form solution to the resource allocation problem. Simulation results showed that the proposed algorithm converged fast, and the NOMA-MEC scheme outperformed the existing orthogonal multiple access (OMA) scheme.

## 1. Introduction

With fifth-generation (5G) networks being available now, the sixth-generation (6G) wireless network is currently under research, which is expected to provide superior performance to satisfy the growing demands of mobile equipment, such as latency-sensitive, energy-hungry, and computationally intensive services and applications [1,2]. For example, the Internet of Things (IoT) networks are being developed rapidly, where massive numbers of nodes are supposed to be connected together, and IoT nodes can not only communicate with each other, but also process acquired data [3,4,5]. However, such IoT and many other terminal devices are constrained by the battery life and computational capability, and thereby, these devices cannot fully support computationally intensive tasks. A conventional approach to improve the computation capability of mobile devices is mobile cloud computing (MCC), where computationally intensive tasks are offloaded to the central cloud servers for data processing [6,7]. However, MCC will cause significant delays due to the long propagation distance between the central server and the user equipment. To address the long transmission delay issue, especially for delay-sensitive applications in the future 6G networks, multi-access edge computing (MEC) has emerged as a decentralized structure to provide the computation capability close to the terminal devices, which is generally implemented at the base stations to provide a cloud-like task processing service [7,8,9,10].

From the communication perspective, non-orthogonal multiple access (NOMA) is recognized as a promising technology to improve the spectral efficiency and massive connectivity, which enable multiple users to utilize the same resource block such as time and frequency for transmission [11,12]. Taking power-domain NOMA as an example, the signals of multiple users are multiplexed in the power domain by superposition coding, and at the receiver side, successive interference cancellation (SIC) is adopted to successively remove the multiple access interference [13]. Hence, integrating NOMA with MEC can potentially improve the service quality of MEC including low transmission latency and massive connections compared to the conventional orthogonal multiple access (OMA).

### 1.1. Related Works

The integration of NOMA and MEC has been well studied so far, and researchers have proposed various approaches to optimal resource allocation to minimize the offloading delay and energy consumption. In [14], the author minimized the offloading latency for a multi-user scenario, in which the power allocation and task partition ratio were jointly optimized. The partial offloading policy can determine the amount of data to be offloaded to the server, and the remainder is processed locally. The author of [15] proposed an iterative two-user NOMA scheme to minimize the offloading latency, in which two users offload their tasks simultaneously by NOMA. Since one of the users suffers performance degradation introduced by NOMA, instead of forcing two users to complete offloading at the same time, the remaining data are offloaded together with the next user during the following time slot. Moreover, many existing works investigated the energy minimization of NOMA-MEC networks. For example, the joint optimization of central processing unit (CPU) frequency, task partition ratio, and power allocation for a NOMA-MEC heterogeneous network were considered in [16,17]. In [18], the author considered a multi-antenna NOMA-MEC network and presented an approach to minimize the weighted sum energy consumption by jointly optimizing the computation and communication resource.

In addition to the existing works on pure NOMA schemes as previously mentioned, a few works also combined NOMA and OMA together, which is denominated as hybrid NOMA [19]. In this paper, the author proposed a two-user hybrid NOMA scenario, in which one user is less delay tolerant than the other. The two users offload during the first time slot by NOMA, and the user with a longer deadline offloads the remaining data during an additional time duration by OMA. This configuration presents significant benefits and outperforms both OMA and pure NOMA in terms of energy consumption since the energy can be saved for the delay-tolerant user instead of finishing offloading at the same time in pure NOMA networks. In [20,21], the hybrid NOMA scheme was extended to multi-user scenarios, in which a two-to-one matching algorithm was utilized to pair every two users into a group. The whole bandwidth resource was divided into multiple sub-channels, and the users in each group offloaded simultaneously through a dedicated sub-channel.

For the resource allocation in NOMA-MEC networks, user grouping is a non-convex problem, which is solved by exhaustive search or applying matching theory. Moreover, deep reinforcement learning (DRL) is recognized as a novel approach to this problem, which is a powerful tool to solve the real-time decision-making tasks, and only a handful papers have utilized it for user grouping and sub-channel assignment, such as [22,23], which output the user grouping policy for uplink and downlink NOMA networks, respectively.

Moreover, in most of the NOMA works, the SIC decoding order is predetermined either by the channel state information (CSI) or the quality of service (QoS) requirements of the users [24,25,26]. A recent work [27] proposed a hybrid SIC scheme to switch the SIC decoding order dynamically, which showed significant performance improvement in uplink NOMA networks. The author of [28] integrated the hybrid SIC scheme with an MEC network to serve two uplink users, and the results revealed that the hybrid SIC outperformed the QoS-based decoding order.

### 1.2. Motivation and Contributions

Motivated by the existing research on MEC-NOMA, in this paper, we investigated the energy minimization for uplink transmission in multi-user hybrid NOMA-MEC networks with hybrid SIC. More specifically, a DRL-based framework was proposed to generate a user grouping policy, and the power allocation, time allocation, and task partition assignment were jointly optimized for each group. The DRL framework collects experience data including CSI, deadlines, and energy consumption as labeled data to train the neural networks (NNs). In association with the resource allocation, the proposed scheme can dynamically adapt the decoding order to achieve better energy efficiency. The main contributions of this paper are summarized as follows:

- A hybrid NOMA-MEC network was proposed, in which an MEC server is deployed at the base station to serve multiple users. All users are divided into pairs, and each pair is assigned into one sub-channel. The users in each group adopt NOMA transmission with the hybrid SIC scheme in the first time duration, and the user with a longer deadline transmits the remaining data by OMA in the following time duration. We proposed a DRL-assisted user grouping framework with joint power allocation, time scheduling, and task partition assignment to minimize the offloading energy consumption under transmission latency and offloading data amount constraints.
- By assuming that the user grouping policy is given, the energy minimization problem for each group is non-convex due to the multiplications of variables and a 0–1 indicator function, which indicates two cases of decoding orders. The solution to the original problem can be obtained by solving each case separately. A multilevel programming method was proposed, where the energy minimization problem was decomposed into three sub-problems including power allocation, time scheduling, and task partition assignment. By carefully analyzing the convexity and monotonicity of each sub-problem, the solutions to all three sub-problems were obtained optimally in closed-form. The solution to the energy minimization problem for each case can be determined optimally by adaptingthe decisions successively from the lower level to the higher level (i.e., from the optimal task partition assignment to the optimal power allocation). Therefore, the solution to the original problem can be obtained by comparing the numerical results of those two cases and selecting the optimal solution with lower energy consumption.
- A DRL framework for user grouping was designed based on a deep Q-learning algorithm. We provided a training algorithm for the NN to learn the experiences based on the channel condition and delay tolerance of each user during a period of slotted time, and the user grouping policy can be learned gradually at the base station by maximizing the negative of the total offloading energy consumption.
- Simulation results are provided to illustrate the convergence speed and the performance of this user grouping policy by comparing with random user grouping policy. Moreover, compared with the OMA-MEC scheme, our proposed NOME-MEC scheme can achieve superior performance with much lower energy consumption.

### 1.3. Organizations

The rest of the paper is structured as follows. The system model and the formulated energy minimization problem for our proposed NOMA-MEC scheme are described in Section 2. Section 3 presents the optimal solution to the energy minimization problem. Following that, the DRL-based user-grouping algorithm is introduced in Section 4. Finally, the simulation results of the convergence and average performance for the proposed scheme are shown in Section 5, and Section 6 concludes this paper.

## 2. System Model and Problem Formulation

### 2.1. System Model

In this paper, we considered a NOMA-MEC network, where a base station is equipped with an MEC server to serve *K* resource-constrained users. During one offloading cycle, each user offloads its task to the MEC server and then obtains the results, which are processed at the MEC server. Generally, the data size of the computation outcome is relatively smaller than the offloaded data in practice; thus, the time for downloading the results can be omitted [18]. Moreover, since the MEC server has a much higher computation capability than the mobile devices, the data processing time at the MEC server can be ignored compared to the offloading time [14]. Therefore, in this work, the total offloading delay was approximated to the time consumption of data uploading to the base station.

We assumed that all *K* users were divided into Φ groups to transmit signals at different sub-channels, and each group ϕ contained two users such that K=2Φ. In each group, we denote the user with a shorter deadline by $U_{m,\phi }$ and the user with relevantly longer deadline by $U_{n,\phi }$, which indicates $\tau_{m,\phi }\leq \tau_{n,\phi }$, where $\tau_{i,\phi }$ is the latency requirement of $U_{i,\phi },\forall i\in \left\{m,n\right\}$ in group ϕ. Because $U_{m,\phi }$ has a tighter deadline, it was assumed that the whole duration $\tau_{m,\phi }$ would be used up, which means that the offloading time $t_{m,\phi }=\tau_{m,\phi }$.

In this paper, we adopted the block channel model, which indicates that the channel condition remains static during each time slot. With the small-scale fading, the channel gain of a user in group ϕ can be expressed as:

(1) $$H_{i,\phi }={\tilde{h}}_{i,\phi }{d_{i,\phi }}^{-\frac{\alpha }{2}},\;\forall i\in \left\{m,n\right\},\forall \phi ,$$

where ${\tilde{h}}_{i,\phi }∼\mathrm{CN}\left(0,1\right)$ is the Rayleigh fading coefficient, $d_{i,\phi }$ is the distance between $U_{i,\phi }$ and the base station, and α is the pass loss exponent. The channel gain is normalized by the addictive white Gaussian noise (AWGN) power with zero mean and $\sigma^{2}$ variance, which can be written as:

(2) $$h_{i,\phi }=\frac{|H_{i,\phi }{|}^{2}}{\sigma^{2}},\;\forall i\in \left\{m,n\right\},\forall \phi .$$

**Figure 1:**
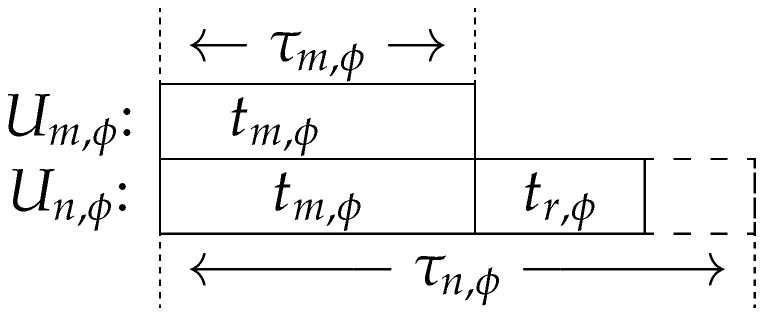
System model.

As shown in Figure 1, since those two users have different delay tolerances, it is natural to consider that $U_{n,\phi }$ is unnecessary to finish offloading within $\tau_{m,\phi }$ via NOMA transmission, and potentially to save energy if $U_{n,\phi }$ can utilize the spare time $\tau_{n,\phi }-\tau_{m,\phi }$. Hence, the adopted hybrid NOMA scheme enables $U_{n,\phi }$ to offload part of its data at the same time when $U_{m,\phi }$ offloads its task during $\tau_{m,\phi }$. An additional time duration $t_{r,\phi }$ is scheduled within each time slot to transmit $U_{n,\phi }$’s remaining data. The task transmission for $U_{n,\phi }$ should be completed within $\tau_{n,\phi }$, which means that $t_{r,\phi }$ should satisfy:

(3) $$t_{r,\phi }\leq \tau_{n,\phi }-\tau_{m,\phi },\forall \phi .$$

As previously mentioned, the users in each group will occupy the same sub-channel to upload their data to the base station simultaneously via NOMA. In NOMA uplink transmission, SIC is adopted at the base station to decode the superposed signal. Conventionally, the SIC decoding order is based on either the user’s CSI or the QoS requirement [27]. For the QoS-based case, to guarantee $U_{m,\phi }$ can offload its data by $\tau_{m,\phi }$, $U_{n,\phi }$ is set to be decoded first, and the achievable rate should satisfy:

(4) $$R_{n,\phi }\leq B\ln\left(1+\frac{P_{n,\phi }{\left|h_{n,\phi }\right|}^{2}}{P_{m,\phi }{\left|h_{m,\phi }\right|}^{2}+1}\right),$$

where *B* is the bandwidth of each sub-channel. $P_{n,\phi }$ and $P_{m,\phi }$ are the transmission power of $U_{n,\phi }$ and $U_{m,\phi }$ during NOMA transmission, respectively. Based on the NOMA principle, the signal of $U_{m,\phi }$ can then be decoded if (4) is satisfied, and the data rate for $U_{m,\phi }$ can be written as:

(5) $$R_{m,\phi }=B\ln\left(1+P_{m,\phi }{\left|h_{m,\phi }\right|}^{2}\right).$$

In contrast, $U_{m,\phi }$ can also be decoded first by treating $U_{n,\phi }$’s signal as interference if the following condition holds:

(6) $$R_{m,\phi }\leq B\ln\left(1+\frac{P_{m,\phi }{\left|h_{m,\phi }\right|}^{2}}{P_{n,\phi }{\left|h_{n,\phi }\right|}^{2}+1}\right).$$

Then, the data rate of $U_{n,\phi }$ can be obtained by removing the information of $U_{m,\phi }$, which is:

(7) $$R_{n,\phi }=B\ln\left(1+P_{n,\phi }{\left|h_{n,\phi }\right|}^{2}\right).$$

If the same power is allocated to $U_{n,\phi }$ for both decoding sequences, it is evident that the achievable rate for $U_{n,\phi }$ in (7) is higher than that in (4) since the interference is removed by the SIC, and hence the decoding order in (7) is preferred in this case. However, since the constraint (6) cannot always be satisfied, the system has to dynamically change the decoding order accordingly to achieve better performance, which motivated us to utilize the hybrid SIC scheme.

In addition, during $t_{r,\phi }$, $U_{n,\phi }$ adopts OMA transmission, and the data rate can be expressed as:

(8) $$R_{r,\phi }=B\ln\left(1+P_{r,\phi }{\left|h_{n,\phi }\right|}^{2}\right),$$

where $P_{r,\phi }$ represents the transmission power of $U_{n,\phi }$ during the second time duration $t_{n,\phi }$.

In this work, the data length of each task is denoted by *L*, which is assumed to be bitwise independent, and we proposed a partial offloading scheme in which each task can be processed locally and remotely in parallel. An offloading partition assignment coefficient $\beta_{\phi }\in \left[0,1\right]$ is introduced, which indicates the amount of data offloaded to the MEC server, and the rest can be executed by the local device in parallel. Thus, for each task, the amount of data for offloading to the server is $\beta_{\phi }L$, and $(1-\beta_{\phi })L$ is the data to be processed locally.

$U_{n,\phi }$ can take the advantage of local computing by executing $(1-\beta_{\phi })L$ data locally during the scheduled NOMA and OMA time duration $t_{m,\phi }+t_{r,\phi }$. Therefore, the energy consumption for $U_{n,\phi }$’s local execution, which is denoted by $E_{n,\phi }^{loc}$, can be expressed as:

(9) $$E_{n,\phi }^{loc}=\frac{\kappa_{0}{\left[C(1-\beta_{\phi })L\right]}^{3}}{{\left(t_{m,\phi }+t_{r,\phi }\right)}^{2}},$$

where $\kappa_{0}$ denotes the coefficient related to the mobile device’s processor and *C* is the number of CPU cycles required for computing each bit.

The total energy consumed by $U_{n,\phi }$ per task involves three parts, including the energy consumed by local computing and transmission during NOMA and OMA offloading. The power for offloading is scheduled separately during these scheduled two time duration according to the hybrid SIC scheme, and thereby, the offloading energy consumption $E_{n,\phi }^{off}$ can be expressed as:

(10) $$E_{n,\phi }^{off}=t_{m,\phi }P_{n,\phi }+t_{r,\phi }P_{r,\phi }.$$

Hence, the total energy consumption can be expressed as:

(11) $$E_{\phi }^{tot}=E_{n,\phi }^{loc}+E_{n,\phi }^{off}.$$

### 2.2. Problem Formulation

We assumed that the resource allocation of $U_{m,\phi }$ is given as a constant in each group since $U_{m,\phi }$ is treated as the primary user whose requirements need to be guaranteed in priority, and we only focused on the energy minimization for $U_{n,\phi }$ during both NOMA and OMA duration. Given the user grouping policy, which will be solved in Section 4, the energy minimization problem for each pair can be formulated as:

(12) $$\begin{array}{cccc} \left(P1\right):\;\min_{\begin{array}{c} P_{n,\phi },P_{r,\phi }\\ t_{r,Œ},{\mathrm{fi}}_{Œ} \end{array}} & \; & \; & \frac{\kappa_{0}{\left[C(1-\beta_{\phi })L\right]}^{3}}{{\left(\tau_{m,\phi }+t_{r,\phi }\right)}^{2}}+\tau_{m,\phi }P_{n,\phi }+t_{r,\phi }P_{r,\phi } \end{array}$$

(13) $$\begin{array}{cccc} s.t. & \; & \; & \tau_{m,\phi }R_{n,\phi }^{H}+t_{r,\phi }B\ln\left(1+P_{r,\phi }{\left|h_{n,\phi }\right|}^{2}\right)\geq \beta_{\phi }L \end{array}$$

(14) $$\begin{array}{cccc} \; & \; & & \tau_{m,\phi }B\ln\left(1+\frac{P_{m,\phi }{\left|h_{m,\phi }\right|}^{2}}{P_{n,\phi }{\left|h_{n,\phi }\right|}^{2}+1}\right)\geq 1_{n,\phi }L \end{array}$$

(15) $$\begin{array}{cccc} \; & \; & & P_{n,\phi }\geq 0,P_{r,\phi }\geq 0 \end{array}$$

(16) $$\begin{array}{cccc} \; & \; & & 0\leq t_{r,\phi }\leq \tau_{n,\phi }-\tau_{m,\phi } \end{array}$$

(17) $$\begin{array}{cccc} \; & \; & & 0\leq \beta_{\phi }\leq 1, \end{array}$$

where $R_{n,\phi }^{H}=1_{n,\phi }B\ln\left(1+P_{n,\phi }{\left|h_{n,\phi }\right|}^{2}\right)+\left(1-1_{n,\phi }\right)B\ln\left(1+\frac{P_{n,\phi }{\left|h_{n,\phi }\right|}^{2}}{P_{m,\phi }{\left|h_{m,\phi }\right|}^{2}+1}\right)$, and $1_{n,\phi }$ is the indicator function. When $1_{n,\phi }=1$, $U_{m,\phi }$ is decoded first, and vice verse. The constraint (13) and (14) ensures all the users should complete offloading of the designated amount of data within the given deadline. The constraint (16) limits that the additionally scheduled time slot should not be beyond $U_{n,\phi }$’s delay tolerance. Constraints (15) and (17) set the feasible range of the transmission power and the offloading coefficient.

The problem (P1) is non-convex due to the multiplication of several variables. Therefore, in the following section, we proposed a multilevel programming algorithm to address the energy minimization problem optimally by obtaining the closed-form solution.

## 3. Energy Minimization for NOMA-MEC with the Hybrid SIC Scheme

In this section, the problem (P1) is solved separately for the case $1_{n,\phi }=1$ and $1_{n,\phi }=0$. Due to the non-convexity of the original problem for both cases, a multilevel programming method is introduced to decompose the problem (P1) into three sub-problems, i.e., power allocation, time slot scheduling, and task assignment, which can be solved optimally by obtaining the closed-form solution. By solving those three sub-problems successively, the optimal solutions for both cases can thereby be obtained, which are provided in the subsections below. The solution to the original problem (P1) can be determined by comparing the numerical result of both cases and choosing the more energy efficient one.

### 3.1. Power Allocation

Let $t_{r,\phi }$ and $\beta_{\phi }$ be fixed. The problem (P1) is regarded as a power allocation problem, which can be rewritten as:

(18) $$\begin{array}{cccc} \left(P2\right):\;\min_{\begin{array}{c} P_{n,\phi },P_{r,\phi } \end{array}} & \; & \; & \frac{\kappa_{0}{\left[C(1-\beta_{\phi })L\right]}^{3}}{{\left(\tau_{m,\phi }+t_{r,\phi }\right)}^{2}}+\tau_{m,\phi }P_{n,\phi }+t_{r,\phi }P_{r,\phi } \end{array}$$

(19) $$\begin{array}{cccc} s.t. & \; & \; & \tau_{m,\phi }R_{n,\phi }^{H}+t_{r,\phi }B\ln\left(1+P_{r,\phi }{\left|h_{n,\phi }\right|}^{2}\right)\geq \beta_{\phi }L \end{array}$$

(20) $$\begin{array}{cccc} \; & \; & & \tau_{m,\phi }B\ln\left(1+\frac{P_{m,\phi }{\left|h_{m,\phi }\right|}^{2}}{P_{n,\phi }{\left|h_{n,\phi }\right|}^{2}+1}\right)\geq 1_{n,\phi }L \end{array}$$

(21) $$\begin{array}{cccc} \; & \; & & P_{n,\phi }\geq 0,P_{r,\phi }\geq 0 \end{array}$$

Since there exists an indicator function, (P2) is solved in two different cases, i.e., when $1_{n,\phi }=1$ and when $1_{n,\phi }=0$. The following theorem provides the optimal solutions of both cases.

**Theorem** **1.** *The optimal power allocation to (P2) is given by the following two cases according to the indicator function:*

*1.*  *For $1_{n,\phi }=1$, $U_{m,\phi }$ is decoded first, and the power allocation for this decoding order is presented in the following three offloading scenarios:*
  *(a)*  *When $P_{n,\phi }\neq 0$ and $P_{r,\phi }\neq 0$, $U_{n,\phi }$ offloads in both time durations, which is termed hybrid NOMA. Given the following two feasible ranges, the optimal power allocation can be expressed as follows:*
    *i.*  *If $P_{m,\phi }>{\left|h_{m,\phi }\right|}^{-2}e^{\frac{\beta_{\phi }L}{B\left(\tau_{m,\phi }+t_{r,\phi }\right)}}\left(e^{\frac{L}{B\tau_{m,\phi }}}-1\right)$,*
      (22) $$P_{n,\phi }^{*}=P_{r,\phi }^{*}={\left|h_{n,\phi }\right|}^{-2}\left(e^{\frac{\beta_{\phi }L}{B\left(\tau_{m,\phi }+t_{r,\phi }\right)}}-1\right).$$
    *ii.*  *If $|h_{m,\phi }{|}^{-2}\left(e^{\frac{L}{B\tau_{m,\phi }}}-1\right)\leq P_{m,\phi }\leq {\left|h_{m,\phi }\right|}^{-2}e^{\frac{\beta_{\phi }L}{B\tau_{m,\phi }}}\left(e^{\frac{L}{B\tau_{m,\phi }}}-1\right)$,*
      (23) $$\begin{array}{cc} \; & P_{n,\phi }^{*}={\left|h_{n,\phi }\right|}^{-2}\left[P_{m,\phi }{\left|h_{m,\phi }\right|}^{2}{\left(e^{\frac{L}{B\tau_{m,\phi }}}-1\right)}^{-1}-1\right], \end{array}$$
      (24) $$\begin{array}{cc} \; & P_{r,\phi }^{*}={\left|h_{n,\phi }\right|}^{-2}\left\{e^{\frac{\beta_{\phi }L}{Bt_{r,\phi }}-\frac{\tau_{m,\phi }}{t_{r,\phi }}\ln\left[P_{m,\phi }{\left|h_{m,\phi }\right|}^{2}{\left(e^{\frac{L}{B\tau_{m,\phi }}}-1\right)}^{-1}\right]}-1\right\}. \end{array}$$
  *(b)*  *When $U_{n,\phi }$ only offloads during the first time duration $\tau_{m,\phi }$, this scheme is termed pure NOMA, and the power allocation is obtained as:*
*if$P_{m,\phi }\geq {\left|h_{m,\phi }\right|}^{-2}e^{\frac{\beta_{\phi }L}{B\tau_{m,\phi }}}\left(e^{\frac{L}{B\tau_{m,\phi }}}-1\right)$,*
    (25) $$P_{n,\phi }^{*}={\left|h_{n,\phi }\right|}^{-2}\left(e^{\frac{\beta_{\phi }L}{B\tau_{m,\phi }}}-1\right).$$
  *(c)*  When $P_{n,\phi }^{*}=0$, $U_{n,\phi }$ chooses to offload solely during the section time duration $t_{r,\phi }$, and the optimal power allocation is:
*if$P_{m,\phi }\geq {\left|h_{m,\phi }\right|}^{-2}\left(e^{\frac{L}{B\tau_{m,\phi }}}-1\right)$,*
    (26) $$P_{r,\phi }^{*}={\left|h_{n,\phi }\right|}^{-2}\left(e^{\frac{\beta_{\phi }L}{Bt_{r,\phi }}}-1\right).$$
*2.*  *For $1_{n,\phi }=0$, $U_{n,\phi }$ is decoded first, and similarly, the power allocation for this decoding order is given in three scenarios:*
  *(a)*  *When $P_{n,\phi }\neq 0$ and $P_{r,\phi }\neq 0$, $U_{n,\phi }$, the hybrid NOMA power allocation is given by:*
*if $P_{m,\phi }\leq {\left|h_{m,\phi }\right|}^{-2}\left(e^{\frac{\beta_{\phi }L}{Bt_{r,\phi }}}-1\right)$,*
    (27) $$\begin{array}{cc} P_{n,\phi }^{*}= & |h_{n,\phi }{|}^{-2}\left(P_{m,\phi }{\left|h_{m,\phi }\right|}^{2}+1\right)\left[e^{\frac{\beta_{\phi }L-t_{r,\phi }B\ln\left(P_{m,\phi }{\left|h_{m,\phi }\right|}^{2}+1\right)}{B\left(\tau_{m,\phi }+t_{r,\phi }\right)}}-1\right] \end{array}$$
    (28) $$\begin{array}{cc} P_{r,\phi }^{*}= & |h_{n,\phi }{|}^{-2}\left[\left(P_{m,\phi }{\left|h_{m,\phi }\right|}^{2}+1\right)e^{\frac{\beta_{\phi }L-t_{r,\phi }B\ln\left(P_{m,\phi }{\left|h_{m,\phi }\right|}^{2}+1\right)}{B\left(\tau_{m,\phi }+t_{r,\phi }\right)}}-1\right]. \end{array}$$
  *(b)*  *When $P_{r,\phi }=0$, the pure NOMA case can be obtained as:*
    (29) $$P_{n,\phi }^{*}={\left|h_{n,\phi }\right|}^{-2}\left(P_{m,\phi }{\left|h_{m,\phi }\right|}^{2}+1\right)\left(e^{\frac{\beta_{\phi }L}{B\tau_{m,\phi }}}-1\right).$$
  *(c)*  When $P_{n,\phi }^{*}=0$, the OMA case is the same as (26).

**Proof.**  Refer to Appendix A.    □

**Remark** **1.** *Theorem 1 provides the optimal power allocation for both decoding sequences, i.e., $U_{m,\phi }$ is decoded first when $1_{n,\phi }=1$, and $U_{n,\phi }$ is decoded first when $1_{n,\phi }=0$. The optimal solution to (P1) is obtained by a numerical comparison between these two cases in terms of energy consumption. Both cases can be further divided into three offloading scenarios including hybrid NOMA, pure NOMA, and OMA based on different power allocations. For the hybrid NOMA case, $U_{n,\phi }$ transmits during both $\tau_{m,\phi }$ and $t_{r,\phi }$, which indicates $P_{n,\phi }>0$, $P_{r,\phi }>0$, and $t_{r,\phi }>0$. The pure NOMA scheme indicates that $U_{n,\phi }$ only transmits simultaneously with $U_{m,\phi }$ during $\tau_{m,\phi }$, and therefore, $P_{r,\phi }=0$ and $t_{r,\phi }=0$. In addition, the OMA case represents that $U_{m,\phi }$ occupies $\tau_{m,\phi }$ solely, and $U_{n,\phi }$ only transmits during $t_{r,\phi }$.*

**Remark** **2.** *Appendix A provides the proof for the case $1_{n,\phi }=1$. The proof for the case $1_{n,\phi }=0$ similarly, and it can be referred to the previous work in [21]. Thus, the proof for the case $1_{n,\phi }=0$ is omitted for this and the following two sub-problems.*

In this subsection, the optimal power allocation for the hybrid NOMA scheme is obtained when $t_{r,\phi }$ is fixed, and then, the optimization of $t_{r,\phi }$ is further studied to minimize $E_{n,\phi }^{tot}$ in the following subsection.

### 3.2. Time Scheduling

The aim of this subsection is to find the optimal time allocation for the second time duration $t_{r,\phi }$, which is solely utilized by $U_{n,\phi }$ for OMA transmission. As previously mentioned in Theorem 1, the optimal power allocation for the hybrid NOMA scheme is given as a function of $t_{r,\phi }$ and $\beta_{\phi }$. Hence, by fixing $\beta_{\phi }$, (P1) is rewritten as:

(30) $$\begin{array}{cccc} \left(P3\right):\;\min_{t_{r,\phi }} & \; & \; & \frac{\kappa_{0}{\left[C(1-\beta_{\phi })L\right]}^{3}}{{\left(\tau_{m,\phi }+t_{r,\phi }\right)}^{2}}+\tau_{m,\phi }P_{n,\phi }^{*}+t_{r,\phi }P_{r,\phi }^{*} \end{array}$$

(31) $$\begin{array}{cccc} s.t.\; & \; & & 0\leq t_{r,\phi }\leq \tau_{n,\phi }-\tau_{m,\phi } \end{array}$$

**Proposition** **1.** *The offloading energy consumption (30) is monotonically decreasing with respected to $t_{r,\phi }$ for both the $1_{n,\phi }=1$ and $1_{n,\phi }=0$ cases. To minimize the energy consumption, the optimal time allocation is to schedule the entire available time before the deadline $\tau_{n,\phi }$, i.e.,*

(32) $$t_{r,\phi }^{*}=\tau_{n,\phi }-\tau_{m,\phi }$$

**Proof.**  Refer to Appendix B.    □

By assuming all the data are offloaded to the MEC server, the following lemma studies the uplink transmission energy efficiency of the two hybrid NOMA-MEC schemes for $1_{n,\phi }=0$ and $1_{n,\phi }=1$.

**Lemma** **1.** *Assume all data are offloaded to the MEC server, i.e., $\beta_{\phi }=1$. The solution in (27) and (28) for the case $1_{n,\phi }=0$ has higher energy consumption than the solution in (22) for the case $1_{n,\phi }=1$, if $|h_{m,\phi }{|}^{-2}\left(e^{\frac{L}{B\tau_{m,\phi }}}-1\right)\leq P_{m,\phi }\leq {\left|h_{m,\phi }\right|}^{-2}\left(e^{\frac{L}{B\left(\tau_{n,\phi }-\tau_{m,\phi }\right)}}-1\right)$.*

**Proof.**  Without considering local computing, the energy consumption for (22) can be written as:

(33) $$E_{1}=\tau_{n,\phi }{\left|h_{n,\phi }\right|}^{-2}\left(e^{\frac{L}{B\tau_{n,\phi }}}-1\right),$$

and the energy consumption for the power allocation scheme in (27) and (28) is given as:

(34) $$\begin{array}{cc} E_{2}= & \tau_{m,\phi }{\left|h_{n,\phi }\right|}^{-2}\left(P_{m,\phi }{\left|h_{m,\phi }\right|}^{2}+1\right)\left[e^{\frac{L-\left(\tau_{n,\phi }-\tau_{m,\phi }\right)B\ln\left(P_{m,\phi }{\left|h_{m,\phi }\right|}^{2}+1\right)}{B\tau_{n,\phi }}}-1\right]\\ & +\left(\tau_{n,\phi }-\tau_{m,\phi }\right){\left|h_{n,\phi }\right|}^{-2}\left[\left(P_{m,\phi }{\left|h_{m,\phi }\right|}^{2}+1\right)e^{\frac{L-\left(\tau_{n,\phi }-\tau_{m,\phi }\right)B\ln\left(P_{m,\phi }{\left|h_{m,\phi }\right|}^{2}+1\right)}{B\tau_{n,\phi }}}-1\right]. \end{array}$$

To prove that $E_{2}\geq E_{1}$, the inequality can be rearranged as:

(35) $$-\tau_{m,\phi }P_{m,\phi }{\left|h_{m,\phi }\right|}^{2}+\tau_{n,\phi }e^{\frac{L}{B\tau_{n,\phi }}}{\left(P_{m,\phi }{\left|h_{m,\phi }\right|}^{2}+1\right)}^{\frac{\tau_{m,\phi }}{\tau_{n,\phi }}}\geq \tau_{n,\phi }e^{\frac{L}{B\tau_{n,\phi }}}.$$

Define $\zeta \left(x\right)=-\tau_{m,\phi }x+\tau_{n,\phi }e^{\frac{L}{B\tau_{n,\phi }}}{\left(x+1\right)}^{\frac{\tau_{m,\phi }}{\tau_{n,\phi }}}$. The first-order derivative of ζ(x) is given as:

(36) $$\zeta^{{}^{\prime }}\left(x\right)=-\tau_{m,\phi }+\tau_{m,\phi }e^{\frac{L}{B\tau_{n,\phi }}}{\left(x+1\right)}^{\frac{\tau_{m,\phi }}{\tau_{n,\phi }}-1}.$$

Therefore, $\zeta^{{}^{\prime }}\left(x\right)$ is monotonically decreasing since $\tau_{m,\phi }<\tau_{n,\phi }$, and the following inequality holds:

(37) $$\zeta^{{}^{\prime }}\left(x\right)\geq \zeta^{{}^{\prime }}\left(e^{\frac{L}{B\left(\tau_{n,\phi }-\tau_{m,\phi }\right)}}-1\right)=0.$$

Hence, for $0\leq x\leq e^{\frac{L}{B\left(\tau_{n,\phi }-\tau_{m,\phi }\right)}}-1$, ζ(x) is monotonically increasing, and $\zeta \left(x\right)\geq \zeta \left(0\right)=\tau_{n,\phi }e^{\frac{L}{B\tau_{n,\phi }}}$, which illustrates that $E_{2}\geq E_{1}$.    □

### 3.3. Offloading Task Partition Assignment

In this subsection, we focused on the optimization of the task partition assignment coefficient for $U_{n.\phi }$ in group ϕ. Given the optimal power allocation and time arrangement, (P1) is reformulated as:

(38) $$\begin{array}{cccc} \left(P4\right):\;\min_{\beta_{\phi }} & \; & & \frac{\kappa_{0}{\left[C(1-\beta_{\phi })L\right]}^{3}}{{\left(\tau_{m,\phi }+t_{r,\phi }^{*}\right)}^{2}}+\tau_{m,\phi }P_{n,\phi }^{*}+t_{r,\phi }^{*}P_{r,\phi }^{*} \end{array}$$

(39) $$\begin{array}{cccc} s.t. & \; & \; & 0\leq \beta_{\phi }\leq 1, \end{array}$$

**Proposition** **2.** *The above problem is convex, and the optimal task assignment coefficient can be characterized by those three optimal power allocation schemes for the hybrid NOMA model in (22), (23), and (27), which is given by:*

(40) $$\beta_{\phi }^{*}=1-\frac{2}{z_{2,\phi }}W\left(\frac{1}{2}z_{1,\phi }^{-\frac{1}{2}}z_{2,\phi }e^{\frac{z_{2,\phi }}{2}}\right),$$

*where W denotes the single-valued Lambert W function and $z_{1,\phi }$ and $z_{2,\phi }$ are determined by the different power allocation schemes, which are presented as follows:*

*(a)*  *$1_{n,\phi }=1$:*
*If (22) is adopted:*
  (41) $$\left\{\begin{array}{cc} \; & z_{1}=\frac{3\kappa_{0}BC^{3}L^{2}{\left|h_{n,\phi }\right|}^{2}}{\tau_{n,\phi }^{2}},\;\\ \; & z_{2}=\frac{L}{B\tau_{n,\phi }} \end{array}\right.$$
*If (23) is adopted:*
  (42) $$\left\{\begin{array}{cc} \; & z_{1}=\frac{3\kappa_{0}B{\left|h_{n,\phi }\right|}^{2}C^{3}L^{2}e^{2u_{\phi }}}{\tau_{n,\phi }^{2}}\\ \; & z_{2}=\frac{L}{B\left(\tau_{n,\phi }-\tau_{m,\phi }\right)} \end{array}\right.$$
  *where $u_{\phi }=\frac{\tau_{m,\phi }}{\left(\tau_{n,\phi }-\tau_{m,\phi }\right)}\ln\left[P_{m,\phi }{\left|h_{m,\phi }\right|}^{2}{\left(e^{\frac{L}{B\tau_{m,\phi }}}-1\right)}^{-1}\right]$.*
*(b)*  *$1_{n,\phi }=0$:*
  (43) $$\left\{\begin{array}{cc} \; & z_{1,\phi }=\frac{3\kappa_{0}BC^{3}L^{2}{\left|h_{n,\phi }\right|}^{2}e^{\frac{\left(\tau_{n,\phi }-\tau_{m,\phi }\right)\ln\left(P_{m,\phi }{\left|h_{m,\phi }\right|}^{2}+1\right)}{\tau_{n,\phi }}}}{{\tau_{n,\phi }}^{2}\left(P_{m,\phi }{\left|h_{m,\phi }\right|}^{2}+1\right)}\\ \; & z_{2,\phi }=\frac{L}{B\tau_{n,\phi }} \end{array}\right.$$

**Proof.**  Refer to Appendix C.    □

**Remark** **3.** *Problem (P4) is the lowest level of the proposed multilevel programming method, which provides three task assignment solutions corresponding to the three power allocation schemes (22), (23), and (27), respectively. The final solution to the energy minimization problem (P1) can be obtained by substituting the optimal task assignment into the corresponding power allocation scheme. Then, the most energy-efficient scheme is selected among (22), (23), and (27) by comparing the numerical energy consumption for each scheme.*

## 4. Deep Reinforcement Learning Framework for User Grouping

In the previous section, it was assumed that the user grouping is given, and the optimal resource allocation was obtained in closed-form. The user grouping can be obtained optimally by exploring all possible user grouping combinations and finding the one with the lowest energy consumption. Although this method provides the optimal user pairing scheme, the complexity of the exhaustive search method is high, and it is not possible to output real-time decisions. Therefore, we proposed a fast converging user pairing training algorithm based on DQN to obtain the user grouping policy, which is introduced in the following subsection, in which the state space, action space, and reward function are defined. Subsequently, the training algorithm for the user grouping policy is provided.

### 4.1. The DRL Framework

The optimization of user grouping is modeled as a DRL task, where the base station is treated as the agent to interact with the environment, which is defined as the MEC network. In each time slot *t*, the agent takes an action $a_{t}$ from the action space A to assign users into pairs according to an optimal policy, which is learned by the DNN. The action taken under current state $s_{t}$ results in an immediate reward $r_{t}$, which is obtained at the beginning of the next time slot, and then moved to the next state $s_{t+1}$. In this problem, the aforementioned terms are defined as follows.

(1) *State space:* The state $s_{t}\in S$ is characterized by the current channel gains and offloading deadlines of all users since the user grouping is mainly determined by those two factors. Therefore, the state $s_{t}$ can be expressed as:
  (44) $$s_{t}=\left\{h_{1}\left[t\right],h_{2}\left[t\right],...,h_{k}\left[t\right],...,h_{K}\left[t\right];\tau_{1}\left[t\right],\tau_{2}\left[t\right],...,\tau_{k}\left[t\right],...,\tau_{K}\left[t\right]\right\}.$$
(2) *Action space:* At each time slot *t*, the agent takes an action $a_{t}\in A$, which contains all the possible user grouping decisions $j_{k,\phi }$. The action is defined as:
  (45) $$a_{t}=\left\{j_{1,1}\left[t\right],...j_{k,\phi }\left[t\right],...j_{K,\Phi }\left[t\right]\right\},$$
  where $j_{k,\phi }=1$ indicates that $U_{k}$ is assigned to group ϕ. In our proposed scheme, two different users can only be assigned to each group.
(3) *Rewards:* The immediate reward $r_{t}$ is described by the sum of the energy consumption of each group after choosing the action $a_{t}$ under state $s_{t}$. The numerical result of the energy consumption in each group can be obtained by solving the problem (P1). Therefore, the reward is defined as:
  (46) $$r_{t}=-\sum_{\phi =1}^{\Phi }E_{\phi }^{tot}\left[t\right]$$

The aim of the agent is to find an optimal policy that maximizes the long-term discounted reward, which can be written as:

(47) $$\begin{array}{cc} R_{t} & =r_{t}+\gamma r_{t+1}+\gamma^{2}r_{t+2}+...\\ \; & =\sum_{i=0}^{\infty }\gamma^{i}r_{t+i}, \end{array}$$

where γ∈[0,1] is the discount factor, which balances the immediate reward and the long-term reward.

### 4.2. DQN-Based NOMA User Grouping Algorithm

**Figure 2:**
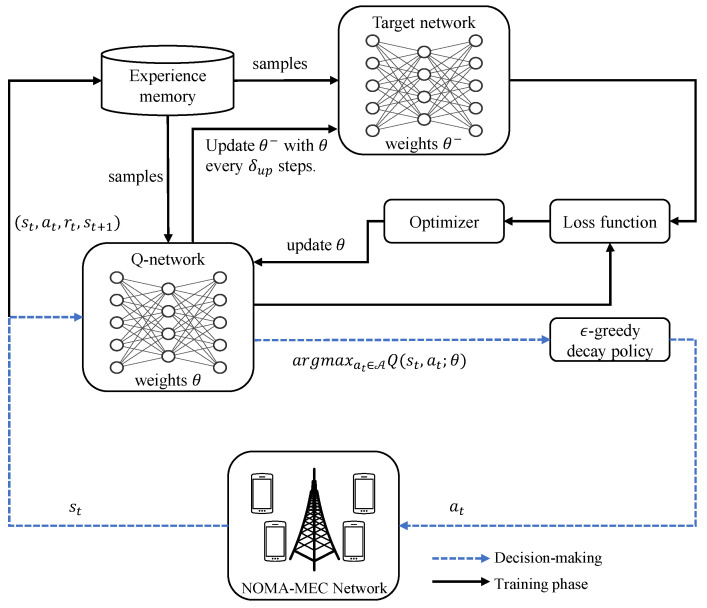
A demonstration of the proposed DQN-based user grouping scheme in the NOMA-MEC network.

To accommodate the reward maximization problem, a DQN-based user-grouping algorithm was proposed in this paper, which is illustrated in Figure 2. In conventional Q-learning, the Q-table is obtained to describe the quality of an action for a given state, and the agent chooses actions according to the Q-values to maximize the reward. However, it will be slow for the system to obtain Q-values for all the state–action pairs if the state space and action space are large. Therefore, to speed up the learning process, instead of generating and processing all possible Q-values, DNNs are introduced to estimate the Q-values based on the weight of DNNs. We utilized a DNN to estimate the Q-value denoted by Q-network, for which the Q-estimation is represented as $Q(s_{t},a_{t};\theta )$, and an additional DNN with the same setting to generate the target network with $Q(s_{t},a_{t};\theta^{-})$ for training, where θ and $\theta^{-}$ are the weights of the DNNs.

We adopted the ϵ-greedy policy with 0<ϵ<1 to balance the exploration of new actions and the exploitation of known actions by either randomly choosing an action $a_{t}\in A$ with probability ϵ to avoid the agent sticking to non-optimal actions or picking the best action with the probability 1−ϵ such that [29]:

(48) $$a_{t}=\arg\max_{a_{t}\in A}Q\left(s_{t},a_{t};\theta \right).$$

Generally, the threshold ϵ is fixed, which indicates that the probability of choosing a random action remains the same throughout the whole training period. However, this brings a fluctuation when the algorithm converges and may lead to divergence again in extreme cases. In this paper, we adopted an ϵ-greedy decay scheme, for which a large $\epsilon^{+}$ (greedier) is given at the beginning, and then, it decays with each training step until a certain small probability $\epsilon^{-}$. The above policy encourages the agent to explore the never-selected actions at the beginning, and then, the agent intends to take more reward-guaranteed actions when the network has already converged.

The target network only updates every certain iteration, which provides a relatively stable label for the estimation network. The agent stores the tuples $\left(s_{t},a_{t},r_{t},s_{t+1}\right)$ as experiences to a memory buffer R, and a mini-batch of samples from the memory is fed into the target network to generate the Q-values labels, which is given by:

(49) $$y_{i}=r_{i}+\max_{a_{i+1}\in A}Q\left(s_{i+1},a_{i+1};\theta^{-}\right),\;\forall i\in R$$

Hence, the loss function for the Q-network can be expressed as:

(50) $$Loss\left(\theta \right)=\left(y_{i}-Q\left(s_{i},a_{i};\theta \right)\right),\;\forall i\in R$$

The Q-network can be trained by minimizing the loss function to obtain the new θ, and the weights of the target network are updated after $\delta_{up}$ steps by replacing $\theta^{-}$ with θ. The whole DQN-based user grouping framework is summarized in Algorithm 1.
**Algorithm 1** DQN-based user-grouping algorithm.1:**Parameter initialization:**2:Initialize Q-network $Q(s_{i},a_{i};\theta )$ and target network $Q(s_{i},a_{i};\theta^{-})$.3:Initialize reply memory R with size |R|, and memory counter.4:Initialize γ, $\epsilon^{+}$, $\epsilon^{-}$, decay step, batch size, target network update interval $\delta_{up}$.5:**Training Phase:**6:**for**$\;episode=1,2,...,N_{ep}$**do**7: **for** $\;time\;step=1,2,...,N_{ts}$ **do**8:  **Input** state $s_{t}$ into Q-network, and obtain Q-values for all actions.9:  Generate a standard uniform distributed random number χ∼U(0,1)10:  **if**  χ>γ  **then**11:   $a_{t}\leftarrow \arg\max_{a_{t}\in A}Q\left(s_{t},a_{t};\theta \right)$12:  **else**13:   Randomly select and action $a_{t}$.14:  **end if**15:  $r_{t}\leftarrow -\sum_{\phi =1}^{\Phi }E_{\phi }^{tot}\left[t\right]$ the observation to next state $s_{t+1}$.16:  Store the experience tuple $\left(s_{t},a_{t},r_{t},s_{t+1}\right)$ into the memory R.17:  **if** memory counter >|R| **then**18:   Remove the old experiences from the beginning.19:  **end if**20:  Randomly sample a mini-batch of the experience tuples $\left(s_{t},a_{t},r_{t},s_{t+1}\right)$ with batch size, and feed into the DNNs.21:  Update the Q-network weights θ by calculating the loss function (50)22:  Update target network weight $\theta^{-}\leftarrow \theta$ every $\delta_{up}$ steps.23: **end for**24:**end for**

## 5. Simulation Results

In this section, several simulation results are presented to evaluate the convergence and effectiveness of the proposed joint resource allocation and user grouping scheme. Specifically, the impact of the learning rate, user number, offloading data length, and delay tolerance is investigated. Moreover, the proposed hybrid SIC scheme is compared to some benchmarks including the QoS-based SIC scheme and other NOMA and OMA schemes.

The system parameters were set up as follows. All users were distributed uniformly and randomly in a disc-shaped cell where the base station was located in the cell center. The total number of users was six, and each of them had a task containing 2 Mbit of data for offloading. As previously mentioned, the delay sensitive primary user $U_{m,\phi }$ was allocated to a predefined power, which was $P_{m,\phi }=0.5$ W for all groups in the simulation. The maximum delay tolerance for each user was 0.25 s. In addition, the rest of the system parameters are listed in Table 1.

To implement the DQN algorithm, the two DNNs were configured with the same settings, where each of them consisted of four fully connected layers, two of which were hidden layers with 200 and 100 neurons, respectively. The activation function we adopted for all hidden layers was the rectified linear unit (ReLU), i.e., f(x)=max(0,x), and the final output layer was activated by tanh, for which the range was (−1,1) [30]. The adaptive moment estimation optimizer (Adam) method was used to learn the DNN weight θ with the given learning rate [31]. The rest of the hyperparameters are listed in Table 2. All simulation results were obtained with PyTorch 1.70 and CUDA 11.1 on the Python 3.8 platform.

### 5.1. Convergence of the Framework

**Figure 3:**
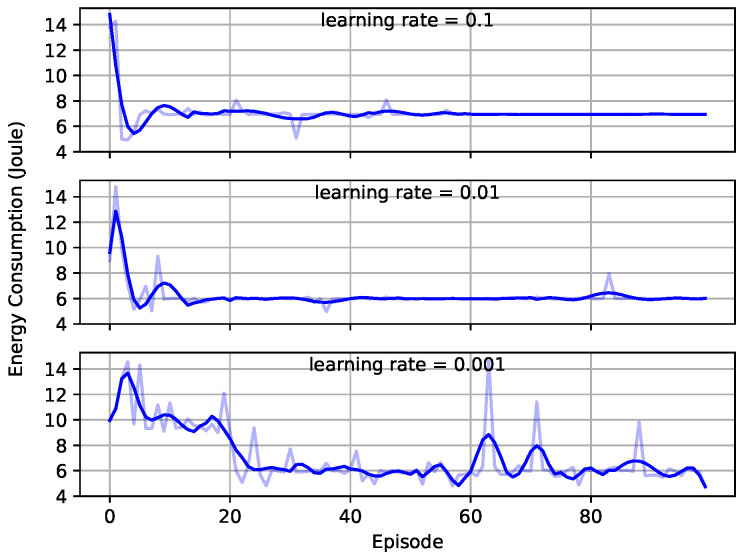
Average energy consumption versus training episodes with different learning rates.

In this part, we evaluated the convergence of the proposed DQN-based user-pairing algorithm. Figure 3 compares the convergence rate of the average reward for each episode under different learning rates, which was described by the average energy consumption. The learning rate controls how much the weights of a DNN based on the network loss should be adjusted, and we set the learning rate =[0.1,0.01,0.001] to observe its influence on the convergence. The network with a 0.1 learning rate converged slightly faster than the one with a 0.01 learning rate, and both of them converged much faster than the network with a 0.001 learning rate. However, when the learning rate was 0.1, even though the higher learning rate had a better convergence, it overshot the minimum and therefore had higher energy consumption after convergence than the other two plots. Moreover, if the learning rate were too low, the network converged slower because it took more episodes to improve the loss function. Therefore, the most suitable learning rate for our proposed DQN network was 0.01, which was adopted to obtain the rest of the simulation results in this paper.

**Figure 4:**
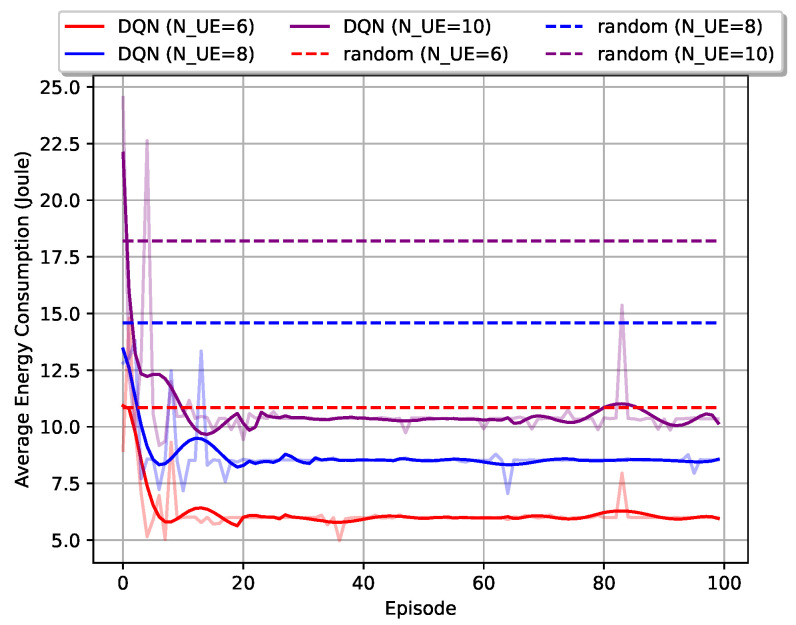
Average energy consumption versus training episodes with different numbers of users.

Figure 4 illustrates the effectiveness of the DQN user-grouping algorithm proposed in this paper. By setting the numbers of users to [6,8,10], the algorithm showed a similar performance that the average energy consumption decreased over training. Although the performance may be worse than the random scheme at the beginning of the training, which was due to the random actions and unstable NN weights, it converged within the first 20 episodes for all three cases. Moreover, more users in the network can result in higher energy consumption, and the algorithm showed superior performance over the random policy, which reduced the energy consumption significantly.

**Figure 5:**
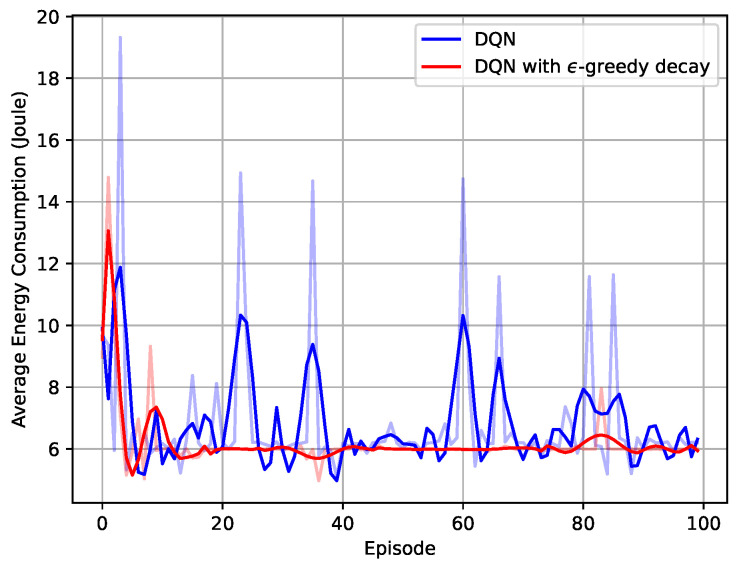
Average energy consumption versus training episodes.

The application of the ϵ-greedy decay policy to the convergence performance is further investigated in Figure 5. The ϵ-greedy coefficient for the blue curve was set to 0.1, which indicated that the probability of the NN to choose a random action was 0.1, and the probability of choosing the action based on (48) was 0.9. The red curve adopted the ϵ-greedy decay policy with the parameters in Table 2. Since the decay policy started with large ϵ, the network was more likely to choose the random action at the beginning, and hence, the energy consumption was higher at the beginning. With ϵ decaying over the episodes, the network chose the actions that were selected before that guaranteed large rewards, and therefore, it was more stable afterwards. Meanwhile, the network without the decay policy had significant fluctuations during training. It had more of a chance to choose the random actions throughout the training, even when the NN had already converged, which may lead to the NN becoming divergent again. However, if a very small ϵ were adopted, the network would be less likely to explore some actions, which may result in being stuck in non-optimal actions.

### 5.2. Average Performance of the Proposed Scheme

**Figure 6:**
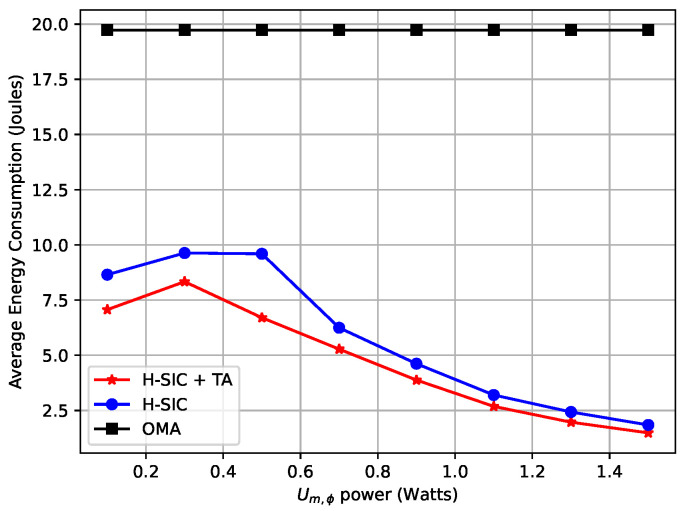
Average energy consumption versus $U_{m,\phi }$’s power.

In this part, we present the average performance of the proposed NOMA-MEC scheme to show the impact of $P_{m,\phi }$, the offloading data length, and the maximum delay tolerance. Meanwhile, our proposed scheme is compared with the one without task assignment and OMA offloading to show the superior performance. As shown in Figure 6, the energy consumption of both hybrid-SIC schemes rose and then decreased as $P_{m,\phi }$ increased. Since $P_{m,\phi }$ was relatively small at the beginning, $U_{m,\phi }$ was not likely to be decoded first to satisfy the constraint (14) in the case $1_{n,\phi }=1$. Therefore, $U_{n,\phi }$ was more likely to be decoded with priority, and increasing $P_{m,\phi }$ caused more interference to $U_{n,\phi }$ according to (4). With $P_{m,\phi }$ continuing to increase, the power allocation schemes in (22) and (23) became feasible, and more groups in the system could adopt different decoding sequences where $U_{m,\phi }$ was decoded first. Then, the energy consumption decreased with the increase of $P_{m,\phi }$, which verified Lemma 1. Moreover, the hybrid-SIC scheme with task assignment outperformed the one without task assignment, shown with the blue line. The one with task assignment had a wider lower bound of the feasible range of power allocation for case $1_{n,\phi }=1$ in (22), which means that it could adopt the $1_{n,\phi }=1$ case with smaller $P_{m,\phi }$. In addition, both hybrid SIC schemes had lower energy consumption than the OMA scheme.

**Figure 7:**
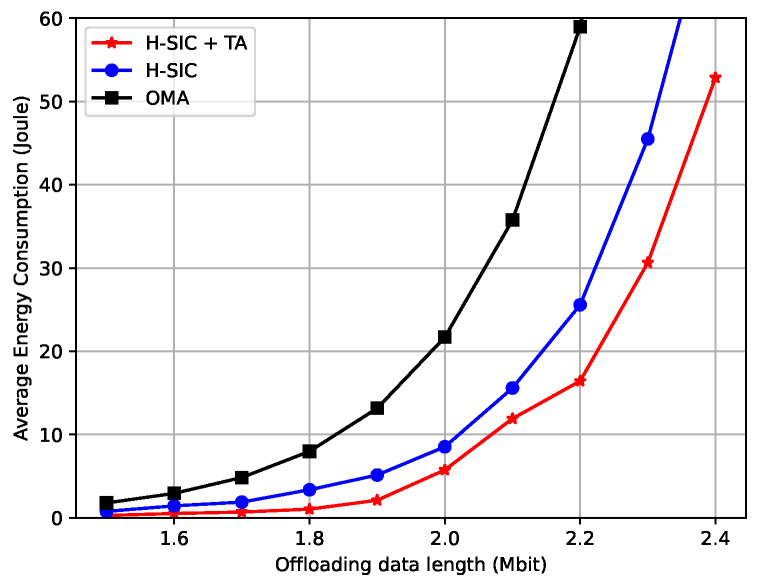
Average energy consumption versus the offloading data length.

**Figure 8:**
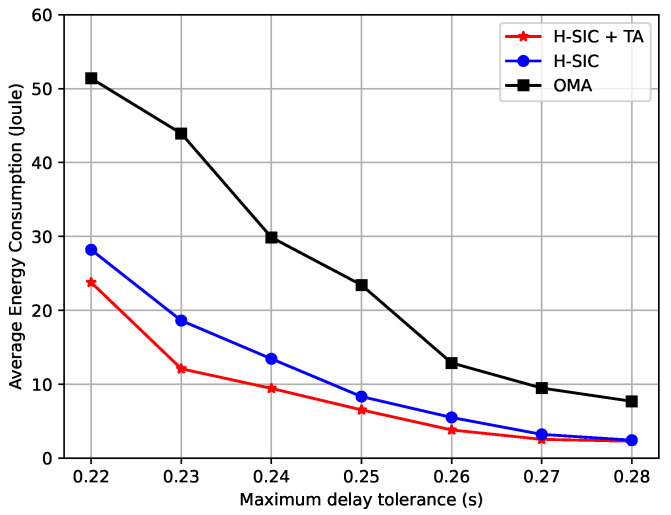
Average energy consumption versus maximum delay tolerance.

In Figure 7, the energy consumption is presented as a function of the offloading data length. As the data length increased, the average energy consumption also grew. Our proposed hybrid-SIC scheme reduced the energy consumption significantly especially when the data length was large. Moreover, Figure 8 reveals the energy consumption comparisons versus the maximum delay tolerance for $U_{n,\phi }$. With tighter deadlines, the energy consumption of the hybrid-SIC scheme was much lower than the OMA scheme, and a greater portion of the data was processed locally to save energy compared to the fully offloaded curve.

## 6. Conclusions

This paper studied the resource allocation problem for a NOMA-assisted MEC network to minimize the energy consumption of users’ offloading activities. The hybrid NOMA scheme had two durations during each time slot, in which NOMA was adopted to serve both users simultaneously during the first time duration, and a dedicated time slot was scheduled to solely offload the remaining part of the more delay-tolerant user by OMA. We assumed the user grouping policy was given at the beginning, the non-convex problem was decomposed into three sub-problems including power allocation, time allocation, and task assignment, which were all solved optimally by studying the convexity and monotonicity. The hybrid SIC scheme selected the SIC decoding order dynamically by the numerical comparison of the energy consumption among different decoding sequences. Finally, after solving those sub-problems, we proposed a DQN-based user-grouping algorithm to obtain the user grouping policy and minimize the long-term average offloading energy consumption. The convergence simulation results showed that the proposed DQN algorithm had similar convergence performance when different numbers of users were chosen, and the ϵ-decay policy was effective at stabilizing the network after convergence. In addition, by comparing with various benchmarks, the partial offloading scheme could reduce the energy consumption compared to full offloading, and the hybrid NOMA transmission outperformed the conventional OMA transmission. Hence, it proved the superiority of the proposed NOMA-MEC scheme in terms of energy consumption.

## Figures and Tables

**Table 1:** System parameters.

Effective capacitance coefficient	$10^{-28}$
Number of CPU cycles required per bit	$10^{3}$
Transmission bandwidth *B*	2 MHz
Path loss exponent α	3.76
Noise spectral density $N_{0}$	−174 dBm/Hz
Maximum cell radius	1000 m
Minimum distance to the base station	50 m

**Table 2:** Hyperparameters.

ϵ-greedy coefficient	0.5–0.01
ϵ-greedy decay steps	2000
Discount factor γ	0.7
Reply memory size R	20,000
Batch size	64
Target network update interval $\delta_{up}$	10
Number of episode $N_{ep}$	100
Number of time steps $N_{ts}$	500

## Data Availability

Not applicable.

## Appendix A. Proof of Theorem 1

By fixing $t_{r,\phi }$ and $\beta_{\phi }$, the above problem in the case $1_{n,\phi }=1$ can be rewritten as:

(A1a) $$\begin{array}{cccc} \left(P5\right):\min_{\begin{array}{c} P_{n,Œ},P_{r,Œ} \end{array}} & \; & \; & \frac{\kappa_{0}{\left[C(1-\beta_{\phi })L\right]}^{3}}{{\left(\tau_{m,\phi }+t_{r,\phi }\right)}^{2}}+\tau_{m,\phi }P_{n,\phi }+t_{r,\phi }P_{r,\phi } \end{array}$$

(A1b) $$\begin{array}{cccc} s.t. & \; & \; & \tau_{m,\phi }B\ln\left(1+P_{n,\phi }{\left|h_{n,\phi }\right|}^{2}\right)+t_{r,\phi }B\ln\left(1+P_{r,\phi }{\left|h_{n,\phi }\right|}^{2}\right)\geq \beta_{\phi }L \end{array}$$

(A1c) $$\begin{array}{cccc} \; & \; & & \tau_{m,\phi }B\ln\left(1+\frac{P_{m,\phi }{\left|h_{m,\phi }\right|}^{2}}{P_{n,\phi }{\left|h_{n,\phi }\right|}^{2}+1}\right)\geq L \end{array}$$

(A1d) $$\begin{array}{cccc} \; & \; & & P_{n,\phi }\geq 0,P_{r,\phi }\geq 0 \end{array}$$

It is evident that the problem is convex, and by rearranging (A1d) as:

(A2) $$\begin{array}{c} P_{n,\phi }|h_{n,\phi }{|}^{2}-P_{m,\phi }{\left|h_{m,\phi }\right|}^{2}{\left(e^{\frac{L}{B\tau_{m,\phi }}}-1\right)}^{-1}+1\leq 0, \end{array}$$

the Lagrangian function can be obtained as follows:

(A3) $$\begin{array}{cc} L(P_{n,\phi },P_{r,\phi },\lambda )= & \frac{\kappa_{0}{\left[C(1-\beta_{\phi })L\right]}^{3}}{{\left(\tau_{m,\phi }+t_{r,\phi }\right)}^{2}}+\tau_{m,\phi }P_{n,\phi }+t_{r,\phi }P_{r,\phi }-\lambda_{1}P_{n,\phi }-\lambda_{2}P_{r,\phi }+\lambda_{3}\beta_{\phi }L\\ \; & -\lambda_{3}\tau_{m,\phi }B\ln\left(1+P_{n,\phi }{\left|h_{n,\phi }\right|}^{2}\right)-\lambda_{3}t_{r,\phi }B\ln\left(1+P_{r,\phi }{\left|h_{n,\phi }\right|}^{2}\right)\\ \; & +\lambda_{4}\left(P_{n,\phi }|h_{n,\phi }{|}^{2}-P_{m,\phi }{\left|h_{m,\phi }\right|}^{2}{\left(e^{\frac{L}{B\tau_{m,\phi }}}-1\right)}^{-1}+1\right), \end{array}$$

where $\lambda ≜[\lambda_{1},\lambda_{2},\lambda_{3},\lambda_{4}]$ are the Lagrangian multipliers. The stationary conditions are given as:

(A4) $$\begin{array}{cc} \; & \frac{\partial L}{\partial P_{n,\phi }}=\tau_{m,\phi }-\lambda_{1}-\lambda_{3}\tau_{m,\phi }B\frac{|h_{n,\phi }{|}^{2}}{P_{n,\phi }{\left|h_{n,\phi }\right|}^{2}+1}+\lambda_{4}{\left|h_{n,\phi }\right|}^{2}=0 \end{array}$$

(A5) $$\begin{array}{cc} \; & \frac{\partial L}{\partial P_{r,\phi }}=t_{r,\phi }-\lambda_{2}-\lambda_{3}t_{r,\phi }B\frac{|h_{n,\phi }{|}^{2}}{P_{r,\phi }{\left|h_{n,\phi }\right|}^{2}+1}=0 \end{array}$$

The Karush–Kuhn–Tucker (KKT) conditions [32] can be obtained as:

(A6) $$\begin{array}{c} \beta_{\phi }L-\tau_{m,\phi }B\ln\left(1+P_{n,\phi }{\left|h_{n,\phi }\right|}^{2}\right)-t_{r,\phi }B\ln\left(1+P_{r,\phi }{\left|h_{n,\phi }\right|}^{2}\right)\leq 0 \end{array}$$

(A7) $$\begin{array}{c} P_{n,\phi }|h_{n,\phi }{|}^{2}-P_{m,\phi }{\left|h_{m,\phi }\right|}^{2}{\left(e^{\frac{L}{B\tau_{m,\phi }}}-1\right)}^{-1}+1\leq 0 \end{array}$$

(A8) $$\begin{array}{c} -P_{n,\phi }\leq 0,-P_{r,\phi }\leq 0 \end{array}$$

(A9) $$\begin{array}{c} \lambda_{i}\geq 0,\;i\in \left\{1,2,3,4\right\} \end{array}$$

(A10) $$\begin{array}{c} \lambda_{1}P_{n,\phi }=0 \end{array}$$

(A11) $$\begin{array}{c} \lambda_{2}P_{r,\phi }=0 \end{array}$$

(A12) $$\begin{array}{c} \lambda_{3}\beta_{\phi }L-\lambda_{3}\tau_{m,\phi }B\ln\left(1+P_{n,\phi }{\left|h_{n,\phi }\right|}^{2}\right)-\lambda_{3}t_{r,\phi }B\ln\left(1+P_{r,\phi }{\left|h_{n,\phi }\right|}^{2}\right)=0 \end{array}$$

(A13) $$\begin{array}{c} \lambda_{4}\left(P_{n,\phi }|h_{n,\phi }{|}^{2}-P_{m,\phi }{\left|h_{m,\phi }\right|}^{2}{\left(e^{\frac{L}{B\tau_{m,\phi }}}-1\right)}^{-1}+1\right)=0 \end{array}$$

(A14) $$\begin{array}{c} \tau_{m,\phi }-\lambda_{1}-\lambda_{3}\tau_{m,\phi }B\frac{|h_{n,\phi }{|}^{2}}{P_{n,\phi }{\left|h_{n,\phi }\right|}^{2}+1}+\lambda_{4}{\left|h_{n,\phi }\right|}^{2}=0 \end{array}$$

(A15) $$\begin{array}{c} t_{r,\phi }-\lambda_{2}-\lambda_{3}t_{r,\phi }B\frac{|h_{n,\phi }{|}^{2}}{P_{r,\phi }{\left|h_{n,\phi }\right|}^{2}+1} \end{array}$$

The power allocation schemes can be obtained by different Lagrangian multipliers decisions as follows:

- Hybrid NOMA: $\lambda_{1}=0$, $\lambda_{2}=0$, and $\lambda_{3}\neq 0$.
  - If $\lambda_{4}=0$:
    (A16) $$P_{n,\phi }^{*}=P_{r,\phi }^{*}={\left|h_{n,\phi }\right|}^{-2}\left(e^{\frac{\beta_{\phi }L}{B\left(\tau_{m,\phi }+t_{r,\phi }\right)}}-1\right)$$
    (A17) $$P_{m,\phi }{\left|h_{m,\phi }\right|}^{2}\geq e^{\frac{\beta_{\phi }L}{B\left(\tau_{m,\phi }+t_{r,\phi }\right)}}\left(e^{\frac{L}{B\tau_{m,\phi }}}-1\right)$$
  - If $\lambda_{4}\neq 0$:
    (A18) $$\begin{array}{cc} \; & P_{n,\phi }^{*}={\left|h_{n,\phi }\right|}^{-2}\left[P_{m,\phi }{\left|h_{m,\phi }\right|}^{2}{\left(e^{\frac{L}{B\tau_{m,\phi }}}-1\right)}^{-1}-1\right], \end{array}$$
    (A19) $$\begin{array}{cc} \; & P_{r,\phi }^{*}={\left|h_{n,\phi }\right|}^{-2}\left\{e^{\frac{\beta_{\phi }L}{Bt_{r,\phi }}-\frac{\tau_{m,\phi }}{t_{r,\phi }}\ln\left[P_{m,\phi }{\left|h_{m,\phi }\right|}^{2}{\left(e^{\frac{L}{B\tau_{m,\phi }}}-1\right)}^{-1}\right]}-1\right\}, \end{array}$$
    where $e^{\frac{\beta_{\phi }L}{B\tau_{m,\phi }}}\left(e^{\frac{L}{B\tau_{m,\phi }}}-1\right)\geq P_{m,\phi }{\left|h_{m,\phi }\right|}^{2}\geq e^{\frac{L}{B\tau_{m,\phi }}}-1$.
- Pure NOMA: $\lambda_{1}=0$, $\lambda_{2}\neq 0$:
  (A20) $$\begin{array}{cc} \; & P_{n,\phi }={\left|h_{n,\phi }\right|}^{-2}\left(e^{\frac{\beta_{\phi }L}{B\tau_{m,\phi }}}-1\right), \end{array}$$
  (A21) $$\begin{array}{cc} \; & P_{r,\phi }=0, \end{array}$$
  where $P_{m,\phi }{\left|h_{m,\phi }\right|}^{2}\geq e^{\frac{\beta_{\phi }L}{B\tau_{m,\phi }}}\left(e^{\frac{L}{B\tau_{m,\phi }}}-1\right)$.
- OMA: $\lambda_{1}\neq 0$, $\lambda_{2}=0$:
  (A22) $$\begin{array}{cc} \; & P_{n,\phi }=0, \end{array}$$
  (A23) $$\begin{array}{cc} \; & P_{r,\phi }={\left|h_{n,\phi }\right|}^{-2}\left(e^{\frac{\beta_{\phi }L}{t_{r,\phi }B}}-1\right). \end{array}$$

## Appendix B. Proof of Proposition 1

The total energy consumption can be expressed as:

(A24) $$\begin{array}{cc} E_{H1}= & \frac{\kappa_{0}{\left[C(1-\beta_{\phi })L\right]}^{3}}{{\left(\tau_{m,\phi }+t_{r,\phi }\right)}^{2}}+\tau_{m,\phi }{\left|h_{n,\phi }\right|}^{-2}\left[P_{m,\phi }{\left|h_{m,\phi }\right|}^{2}{\left(e^{\frac{L}{B\tau_{m,\phi }}}-1\right)}^{-1}-1\right]\\ \; & +t_{r,\phi }{\left|h_{n,\phi }\right|}^{-2}\left\{e^{\frac{\beta_{\phi }L}{Bt_{r,\phi }}-\frac{\tau_{m,\phi }}{t_{r,\phi }}\ln\left[P_{m,\phi }{\left|h_{m,\phi }\right|}^{2}{\left(e^{\frac{L}{B\tau_{m,\phi }}}-1\right)}^{-1}\right]}-1\right\}, \end{array}$$

where

$a_{\phi }=\frac{\beta_{\phi }L-B\tau_{m,\phi }\ln\left[P_{m,\phi }{\left|h_{m,\phi }\right|}^{2}{\left(e^{\frac{L}{B\tau_{m,\phi }}}-1\right)}^{-1}\right]}{B}$.

(A25) $$\begin{array}{c} \frac{\partial E_{H1}}{\partial t_{r,\phi }}=-\frac{2\kappa_{0}{\left[C(1-\beta_{\phi })L\right]}^{3}}{{\left(\tau_{m,\phi }+t_{r,\phi }\right)}^{3}}+{\left|h_{n,\phi }\right|}^{-2}\left(e^{\frac{a_{\phi }}{t_{r,\phi }}}-\frac{a_{\phi }}{t_{r,\phi }}e^{\frac{a_{\phi }}{t_{r,\phi }}}-1\right). \end{array}$$

Define $g\left(x\right)=e^{\frac{a_{\phi }}{x}}-\frac{a_{\phi }}{x}e^{\frac{a_{\phi ,1}}{x}}-1$,

(A26) $$g^{\prime }\left(x\right)=\frac{a_{\phi ,1}^{2}e^{\frac{a_{\phi ,1}}{x}}}{x^{3}}\geq 0,\;\forall x>0.$$

Hence, g(x) is monotonically increasing for x>0, and $g\left(t_{r,\phi }\right)\leq g\left(\infty \right)=0$.

Therefore, $\frac{dE_{H1}}{dt_{r,\phi }}\leq 0$, which is monotonically decreasing. Hence, the larger $t_{r,\phi }$ scheduled, the less energy is consumed, and the optimal situation is when $t_{r,\phi }^{*}=\tau_{n,\phi }-\tau_{m,\phi }$.

For the power allocation scheme in (23), the energy consumption is given as:

(A27) $$\begin{array}{cc} E_{H2}= & \frac{\kappa_{0}{\left[C(1-\beta_{\phi })L\right]}^{3}}{{\left(\tau_{m,\phi }+t_{r,\phi }\right)}^{2}}+\left(\tau_{m,\phi }+t_{r,\phi }\right){\left|h_{n,\phi }\right|}^{-2}\left(e^{\frac{\beta_{\phi }L}{B\left(\tau_{m,\phi }+t_{r,\phi }\right)}}-1\right). \end{array}$$

By obtaining the derivative with respect to $t_{r,\phi }$,

(A28) $$\frac{\partial E_{H2}}{\partial t_{r,\phi }}=-\frac{2\kappa_{0}{\left[C(1-\beta_{\phi })L\right]}^{3}}{{\left(\tau_{m,\phi }+t_{r,\phi }\right)}^{3}}+{\left|h_{n}\right|}^{-2}\left(e^{\frac{\beta_{\phi }L}{B\left(\tau_{m,\phi }+t_{r,\phi }\right)}}-t_{r,\phi }\frac{\beta_{\phi }L}{B\left(\tau_{m,\phi }+t_{r,\phi }\right)}e^{\frac{\beta_{\phi }L}{B\left(\tau_{m,\phi }+t_{r,\phi }\right)}}-1\right).$$

Define $g_{2}\left(x\right)≜e^{\frac{\beta_{\phi }L}{B\left(\tau_{m,\phi }+x\right)}}-x\frac{\beta_{\phi }L}{B\left(\tau_{m,\phi }+x\right)}e^{\frac{\beta_{\phi }L}{B\left(\tau_{m,\phi }+x\right)}}-1$, and the derivative of $g_{2}\left(x\right)$ is:

(A29) $$g_{2}^{\prime }\left(x\right)=\frac{{\left(\beta_{\phi }L\right)}^{2}}{B^{2}{\left(\tau_{m,\phi }+x\right)}^{3}}e^{\frac{\beta_{\phi }L}{B\left(\tau_{m,\phi }+x\right)}}\geq 0,\;\forall x>0.$$

Thus, $g_{2}\left(x\right)$ is monotonically increasing for x>0, and $g\left(t_{r,\phi }\right)\leq g\left(\infty \right)=0$, which indicates $\frac{dE_{H2}}{dt_{r,\phi }}\leq 0$. Similar to the previous case, the energy function is monotonically decreasing with respect to $t_{r,\phi }$, and the optimal time allocation is $t_{r,\phi }^{*}=\tau_{n,\phi }-\tau_{m,\phi }$.

## Appendix C. Proof to Proposition 2

(A30) $$\begin{array}{cccc} \min_{\beta_{\phi }} & \; & & \frac{\kappa_{0}{\left[C(1-\beta_{\phi })L\right]}^{3}}{{\left(\tau_{m,\phi }+t_{r,\phi }^{*}\right)}^{2}}+\tau_{m,\phi }P_{n,\phi }^{*}+t_{r,\phi }^{*}P_{r,\phi }^{*} \end{array}$$

(A31) $$\begin{array}{cccc} s.t. & \; & \; & 0\leq \beta_{\phi }\leq 1. \end{array}$$

The Lagrangian is given as:

(A32) $$L\left(\beta_{\phi },\lambda_{5},\lambda_{6}\right)=\frac{\kappa_{0}{\left[C(1-\beta_{\phi })L\right]}^{3}}{{\left(\tau_{m,\phi }+t_{r,\phi }^{*}\right)}^{2}}+\tau_{m,\phi }P_{n,\phi }^{*}+t_{r,\phi }^{*}P_{r,\phi }^{*}-\lambda_{5}\beta_{\phi }+\lambda_{6}\left(\beta_{\phi }-1\right)$$

- For the case $P_{m,\phi }=P_{n,\phi }$ in (22), the stationary condition is obtained as:
  (A33) $$\frac{\partial L}{\partial \beta_{\phi }}=\frac{-3\kappa_{0}{\left(CL\right)}^{3}{\left(1-\beta_{\phi }\right)}^{2}}{\tau_{n,\phi }^{2}}+\frac{L}{B}{\left|h_{n,\phi }\right|}^{-2}e^{\frac{\beta_{\phi }L}{B\tau_{n,\phi }}}-\lambda_{5}+\lambda_{6}=0.$$
  Therefore, the KKT conditions can be written as follows:
  (A34) $$\begin{array}{c} -\beta_{\phi }\leq 0 \end{array}$$
  (A35) $$\begin{array}{c} \beta_{\phi }-1\leq 0 \end{array}$$
  (A36) $$\begin{array}{c} \lambda_{5}\beta_{\phi }=0 \end{array}$$
  (A37) $$\begin{array}{c} \lambda_{6}\left(\beta_{\phi }-1\right)=0 \end{array}$$
  (A38) $$\begin{array}{c} \frac{-3\kappa_{0}{\left(CL\right)}^{3}{\left(1-\beta_{\phi }\right)}^{2}}{\tau_{n,\phi }^{2}}+\frac{L}{B}{\left|h_{n,\phi }\right|}^{-2}e^{\frac{\beta_{\phi }L}{B\tau_{n,\phi }}}-\lambda_{5}+\lambda_{6}=0 \end{array}$$
  For $\beta_{\phi }>0$, $\lambda_{5}=\lambda_{6}=0$, and (A38) can be rewritten as:
  (A39) $$\frac{3\kappa_{0}{\left(CL\right)}^{3}{\left(1-\beta_{\phi }\right)}^{2}}{\tau_{n,\phi }^{2}}=\frac{L}{B}{\left|h_{n,\phi }\right|}^{-2}e^{\frac{\beta_{\phi }L}{B\tau_{n,\phi }}}.$$
  Define $z_{1,\phi }=\frac{3\kappa_{0}BC^{3}L^{2}{\left|h_{n,\phi }\right|}^{2}}{\tau_{n,\phi }^{2}}$, $z_{2,\phi }=\frac{L}{B\tau_{n,\phi }}$, and $b_{\phi }=\left(1-\beta_{\phi }\right)$. The optimal task assignment coefficient can be derived as:
  (A40) $$z_{1,\phi }b_{\phi }^{2}=e^{z_{2,\phi }\left(1-b_{\phi }\right)},$$
  (A41) $$b_{\phi }=\frac{2}{z_{2,\phi }}W\left(\frac{1}{2}z_{1,\phi }^{-\frac{1}{2}}z_{2,\phi }e^{\frac{z_{2,\phi }}{2}}\right).$$
  The optimal task assignment ratio can be expressed as:
  (A42) $$\beta_{\phi }^{*}=1-b=1-\frac{2}{z_{2,\phi }}W\left(\frac{1}{2}z_{1,\phi }^{-\frac{1}{2}}z_{2,\phi }e^{\frac{z_{2,\phi }}{2}}\right).$$
- For the case $P_{m,\phi }\neq P_{n,\phi }$ in (23):
  The stationary condition can be expressed as:
  (A43) $$\frac{\partial L}{\partial \beta_{\phi }}=\frac{-3\kappa_{0}{\left(CL\right)}^{3}{\left(1-\beta_{\phi }\right)}^{2}}{\tau_{n,\phi }^{2}}+{\left|h_{n,\phi }\right|}^{-2}\frac{L}{B}e^{-u}e^{\frac{\beta_{\phi }L}{B\left(\tau_{n,\phi }-\tau_{m,\phi }\right)}-u}-\lambda_{5}+\lambda_{6}=0,$$
  where $u_{\phi }=\frac{\tau_{m,\phi }}{\left(\tau_{n,\phi }-\tau_{m,\phi }\right)}\ln\left[P_{m,\phi }{\left|h_{m,\phi }\right|}^{2}{\left(e^{\frac{L}{B\tau_{m,\phi }}}-1\right)}^{-1}\right]$.
  (A44) $$\begin{array}{c} -\beta_{\phi }\leq 0 \end{array}$$
  (A45) $$\begin{array}{c} \beta_{\phi }-1\leq 0 \end{array}$$
  (A46) $$\begin{array}{c} \lambda_{5}\beta_{\phi }=0 \end{array}$$
  (A47) $$\begin{array}{c} \lambda_{6}\left(\beta_{\phi }-1\right)=0 \end{array}$$
  (A48) $$\begin{array}{c} \frac{-3\kappa_{0}{\left(CL\right)}^{3}{\left(1-\beta_{\phi }\right)}^{2}}{\tau_{n,\phi }^{2}}+{\left|h_{n,\phi }\right|}^{-2}\frac{L}{B}e^{-u_{\phi }}e^{\frac{\beta_{\phi }L}{B\left(\tau_{n,\phi }-\tau_{m,\phi }\right)}-u_{\phi }}-\lambda_{5}+\lambda_{6}=0 \end{array}$$
  For $\beta_{\phi }>0$, $\lambda_{5}=\lambda_{6}=0$, constraint (A48) can be rearranged as:
  (A49) $$\frac{3\kappa_{0}{\left(CL\right)}^{3}{\left(1-\beta_{\phi }\right)}^{2}}{\tau_{n,\phi }^{2}}={\left|h_{n,\phi }\right|}^{-2}\frac{L}{B}e^{-u_{\phi }}e^{\frac{\beta_{\phi }L}{B\left(\tau_{n,\phi }-\tau_{m,\phi }\right)}-u_{\phi }},$$
  (A50) $$\frac{3\kappa_{0}B{\left|h_{n,\phi }\right|}^{2}{\left(CL\right)}^{3}e^{2u_{\phi }}{\left(1-\beta_{\phi }\right)}^{2}}{\tau_{n,\phi }^{2}L}=e^{\frac{\beta_{\phi }L}{B\left(\tau_{n,\phi }-\tau_{m,\phi }\right)}}.$$
  Define $z_{1,\phi }=\frac{3\kappa_{0}B{\left|h_{n,\phi }\right|}^{2}C^{3}L^{2}e^{2u_{\phi }}}{\tau_{n,\phi }^{2}}$, $z_{2,\phi }=\frac{L}{B\left(\tau_{n,\phi }-\tau_{m,\phi }\right)}$. The above equation can be rewritten as:
  (A51) $$z_{1,\phi }b_{\phi }^{2}=e^{z_{2,\phi }\left(1-b_{\phi }\right)},$$
  (A52) $$b_{\phi }=\frac{2}{z_{2,\phi }}W\left(\frac{1}{2}z_{1,\phi }^{-\frac{1}{2}}z_{2,\phi }e^{\frac{z_{2,\phi }}{2}}\right),$$
  Hence, the optimal task partition assignment ratio is:
  (A53) $$\beta_{\phi }^{*}=1-b_{\phi }=1-\frac{2}{z_{2,\phi }}W\left(\frac{1}{2}z_{1,\phi }^{-\frac{1}{2}}z_{2,\phi }e^{\frac{z_{2,\phi }}{2}}\right).$$
